# Exploring the differential effects of career and psychosocial mentoring on newcomer socialization

**DOI:** 10.3389/fpsyg.2022.975064

**Published:** 2022-12-02

**Authors:** Hui Deng, Wenbing Wu, Yihua Zhang, Zhuyan Yu, Hanzhi Xu, Wen Wu

**Affiliations:** ^1^School of Economics and Management, Beijing Jiaotong University, Beijing, China; ^2^Graduate School of Education and Psychology, Pepperdine University, Los Angeles, CA, United States; ^3^School of Social Policy and Practice, University of Pennsylvania, Philadelphia, PA, United States; ^4^School of Music and Recording Arts, Communication University of China, Beijing, China

**Keywords:** mentoring, self-efficacy, learning adaptability, newcomer socialization, social cognitive career theory

## Abstract

Drawing on the social cognitive career theory, this study proposed an integrative framework to uncover how and when different types of mentoring accelerate newcomer’s socialization in corresponding domains. We tested this relational model with time-lagged, multisource survey data collected from 157 newcomers and 88 supervisors. The results indicated that career mentoring facilitated newcomer task mastery, task performance, and job satisfaction by improving newcomer occupational self-efficacy, whereas psychosocial mentoring promoted newcomer job satisfaction and social integration *via* inspiring newcomer social self-efficacy. Furthermore, newcomer learning adaptability amplified the influence of career mentoring on newcomer occupational self-efficacy, as well as the impact of psychosocial mentoring on newcomer social self-efficacy. Our study extended the mentoring and socialization literature and provided significant practical implications for managers on how to arrange tailored mentoring to facilitate newcomer socialization.

## Introduction

Organizational socialization is the process by which individuals acquire the essential knowledge and social skills to perform their organizational roles (Van Maanen and Schein, 1979; Bauer et al., 2007). The adequate transition of new hires from organizational outsiders to insiders contributes to their positive outcomes, including organizational commitment, job involvement, and organizational citizenship behavior (Madlock and Chory, 2014; Adil et al., 2021), which can further help organizations flourish and successfully achieve optimal organizational performance (Adil et al., 2021). Given the instrumental roles of socialized newcomers in achieving organizational objectives, supervisors or veterans tend to proactively or passively provide various supports to guide them to socialize. These incumbents become important sources of support to facilitate newcomer’s transition into the organizations (Kammeyer-Mueller and Wanberg, 2003; Chong et al., 2021; Zheng et al., 2021). As such, mentoring, an important organizational tool to facilitate the socialization process provided by these experienced organizational employees, has garnered increasing attention from scholars focused on newcomer adjustment.

Mentoring refers to “a process for the informal transmission of knowledge, social capital, and psychosocial support perceived by the recipients as relevant to work, career, or professional development” (Bozeman and Feeney, 2007, p. 731). Such transmission often occurs from senior colleagues with advanced work experiences (e.g., supervisors or veterans) to junior colleagues (e.g., new hires) and contains two functions: career and psychosocial mentoring (Kocha, 2017; Mullen and Klimaitis, 2021). Career mentoring focuses on providing task-related aspects of support to mentees’ work, involving sponsorship, coaching, exposure, and opportunities in order to enhance their job-related knowledge and skills (Allen et al., 2004; Kao et al., 2020), whereas psychosocial mentoring reflects its social-psychological functions, underlining providing counseling, friendship, acceptance, and confirmation (Kammeyer-Mueller and Judge, 2008; Cheung et al., 2022).

To date, numerous scholars have bridged the socialization and mentoring literature, and most of them focus on revealing mentoring functions in facilitating socialization (Son, 2016; Cai et al., 2021; Zheng et al., 2021). Nevertheless, largely neglected in this line of research is how and when the aforementioned two forms of mentoring differentially promote newcomers to manage socialized periods with certain outcomes (Yang et al., 2013). Clarifying this issue is essential and worthy because by mentoring newcomers in accordance with their shortfalls in socialization, organizational resources can be properly and effectively arranged (Mathews, 2003; Carter and Youssef-Morgan, 2019). For newcomers, it also can be conducive to allocating limited personal energy to develop certain mentorships (Shamblen et al., 2020), which, in turn, facilitates the socialization process catering to their elastic individualized needs. Our study, therefore, focuses on exploring the effects of different types of informal mentoring that newcomers receive on their socialization. Furthermore, as organizational socialization is an essential initiator and mentoring serves as an important facilitator of employee career management (Fang et al., 2011; Van Vianen et al., 2018), we suggest that the social cognition career theory may provide a suitable framework to explain the aforementioned complex effects.

The social cognitive career theory posits that contextual factors of supports or barriers can facilitate or inhibit employee career goals through taking part in the formulation of their person-cognitive variables (Lent et al., 2002). In the context of newcomer adjustment, developing specific mentorships aims to improve newcomers’ abilities and skills to master new environment in corresponding domains (Ghosh, 2014). Accordingly, specific self-efficacy, reflecting individuals’ self-cognition of their abilities in certain fields (Schwoerer et al., 2005; Zhao et al., 2021), may be a potential mechanism to explain the relationship between different types of mentoring and newcomer socialization outcomes. Specifically, we propose that career and psychosocial mentoring can be regarded as contextual supports that impose significant influences on mentees certain learning experiences (Craig et al., 2013; Yang et al., 2013) and then promote their self-efficacy in specific domains (i.e., occupational and social). Furthermore, given that individuals’ senses of self-efficacy in the specific field determine how much effort they will execute into that field and what they will achieve accordingly (Bandura, 2006), employees with high domain-specific self-efficacy are prone to successfully socialize in the corresponding field, resulting in certain outcomes (Lent et al., 2000; Luo et al., 2018; Tomas et al., 2019). Thus, we speculate that career and psychosocial mentoring will act as contextual supports to facilitate newcomer socialization outcomes in occupational (e.g., task mastery, task performance, and job satisfaction) and social (e.g., job satisfaction and social integration) domains through stimulating their occupational and social self-efficacy, respectively.

Furthermore, it has been well documented that learning can be regarded as the main process leading to effective mentoring and socialization as it especially determines whether inexperienced mentees acquire knowledge and skills and whether newcomers are successfully socialized (Allen et al., 2017; Nasr et al., 2019). Combining the argument from the social cognitive career theory that individuals’ characteristics can affect their learning process (Lent et al., 1994; Lopez et al., 1997), we thus propose that newcomers’ learning adaptability, an individual characteristic closely relevant to their learning experience, may serve as a potential moderator in the relationship between mentoring and newcomers’ self-efficacy. Learning adaptability refers to an employee’s willingness to adjust to the learning aspects of the new skills, tasks, and situations (Ployhart and Bliese, 2006). Mentees with high learning adaptability tend to be skilled in learning and absorbing the external information provided by their mentors (Wang et al., 2011) and internalizing it to adjust their cognitive structures, which can facilitate them to develop self-cognition (Lent et al., 2002). Thus, newcomer learning adaptability is introduced as a boundary condition to moderate the linkages between career and psychosocial mentoring and newcomer occupational and social self-efficacy. In summary, our study applies the social cognitive career theory to illustrate the underlying mechanisms and boundary conditions of the differential links between career and psychosocial mentoring and newcomer task-related and social outcomes with occupational and social self-efficacy as mediators and learning adaptability as a moderator.

Our research contributes to mentoring and socialization literature from the following three aspects. First, our study extends Yang et al.’s (2013) work and provides a more nuanced understanding of bridging the socialization and mentoring literature by exploring how career and psychosocial mentoring differentially affect new hire’s corresponding socialization outcomes. Second, drawing on the social cognitive career theory, our study incorporates domain-specific self-efficacy as the pivotal person-cognitive mechanism in linking different types of mentoring and newcomer task-related and social outcomes. We also answer Allen et al.’s (2017) call to advance the understanding of when mentoring facilitates some content areas of socialization. Third, our study offers a nuanced explanation of differences in mentees’ self-efficacy improvement degree after receiving mentoring from the perspective of mentee learning features, whereby contending that mentees’ learning adaptability is a boundary condition for the aforementioned relationships.

## Theoretical background and research hypotheses

### Newcomers’ mentoring and self-efficacy

Self-efficacy refers to individuals’ beliefs in their capabilities to produce given attainments by exercising influence over events associated with their lives (Bandura, 1977). Given that people differ in domains and degree to which they develop their self-efficacy, scholars differentiated a set of self-beliefs in the different contexts, including but not limited to occupational self-efficacy, social self-efficacy, academic self-efficacy, and creative self-efficacy (Schyns and von Collani, 2002; Fan et al., 2013; Loeb et al., 2016; Wang et al., 2018; Mazzetti et al., 2020). Extensive research on self-efficacy emphasized its potential to predict a host of positive outcomes, such as job satisfaction, career commitment, subjective career success, and academic achievement (cf. Niu, 2010; Ozyilmaz et al., 2018; Mazzetti et al., 2020; Rigotti et al., 2020). Specifically, Bandura (2006) indicated that utilizing one’s domain-specific self-efficacy within the corresponding context in linking contextual factors to relevant job-related outcomes may be better than using generalized self-efficacy. Therefore, as the settings considered in our study are career and psychosocial mentoring, which focus on occupational and social supports in the workplace, respectively, occupational and social self-efficacy beliefs will be examined. In particular, occupational self-efficacy captures an individual’s sense of confidence in his or her capability to successfully master tasks (Schyns and von Collani, 2002), while social self-efficacy reflects the confidence in his or her capability to engage in the social interactional tasks that are necessary to develop and maintain interpersonal relationships (Smith and Betz, 2000).

The social cognitive career theory stresses that contextual supports could promote employees’ certain learning experiences and further affect their self-efficacy (Lent et al., 2002); it thus may provide a theoretical framework to explore the link between specific mentoring and domain-specific self-efficacy. As Craig et al. (2013) and Cheung et al. (2022) suggested, career and psychosocial mentoring can serve as contextual supports from more-experienced senior colleagues for employees since they are infused with several useful and distinct environmental information and work experiences. Thus, we anticipated that external supports in the forms of these two kinds of mentoring could increase newcomers’ domain-specific self-efficacy beliefs by influencing their certain learning experiences (Kao et al., 2019). Specifically, combined with social cognitive mechanisms, newcomers who have received career mentoring may internalize these task-related skills and knowledge during the learning process and then form the cognition that these successful learning experiences provide evidence for the improvement in their professional work ability (Williams and Subich, 2006; Lent et al., 2010). Finally, such positive cognition may increase their occupational self-efficacy (Međugorac et al., 2020). In a similar vein, we suggest that receiving psychosocial mentoring may increase newcomers’ knowledge and skills related to interpersonal interaction and psychological state, such as feasible solutions to interpersonal conflicts, how to develop favorable relationships with other group members, how to understand others and control interpersonal interactions at work, and how to alleviate work-related stress (Raabe and Beehr, 2003; Greiman, 2007; Craig et al., 2013; Chiesa et al., 2019). These successful learning experiences in the social domain may further help newcomers to generate confidence in maintaining pleasurable interpersonal relationships and know social self-efficacy (Međugorac et al., 2020).

In addition, previous research on self-efficacy has also highlighted that mentoring played a crucial role in building employee self-efficacy beliefs through vicarious learning and verbal persuasion (Hout, 2013; Sheu et al., 2018). Specifically, receiving mentoring not only can provide employees the opportunity to observe mentors performing the desired tasks with specific talents and rehearse their skills subsequently but also provides another opportunity for mentors to persuade employees that they have the personal ability to complete the task. As a result, it is conceivable that newcomers who have received career or psychosocial mentoring are likely to obtain relevant work experiences as well as master professional skills and receive positive hints about their personal abilities from mentors, which help them to construct positive cognitive appraisals of domain-specific self-efficacy. Based on the previous discussion, we thus assume:

*Hypothesis 1: Newcomer career mentoring is positively related to occupational self-efficacy.*

*Hypothesis 2: Newcomer psychosocial mentoring is positively related to social self-efficacy.*

### Newcomers’ self-efficacy and socialization outcomes

The social cognitive career theory proposes that individuals’ self-efficacy further affects their choices or goals to conduct particular career-related activities (Lent et al., 2000). That is, employees who have high levels of domain-specific self-efficacy beliefs are likely to execute courses of action in the given field, resulting in certain career outcomes (Međugorac et al., 2020). This coincides with Bandura’s (1984) proposition that individuals’ self-efficacy beliefs affect not only the initiation of behavior, the investment of effort and energy, and the persistence in the fight against difficulties but also the achievement of given goals. Thus, according to the social cognitive career theory, we speculate that newcomers who possess high occupational and social self-efficacy may be inclined to exert the effort required to overcome obstacles and to cope with entry anxiety and uncertainty, respectively, resulting in their successful socialization and adjustment in occupational and social domains. Moreover, existing research on mentoring has stated that the impacts of receiving mentoring can be continuous, which can induce short- and long-term benefits for mentees, including increasing emotional support, wellbeing, and career success (Liu et al., 2020; Cai et al., 2021). Our study thus takes into account both typical proximal benefits (task mastery and social integration) and distal outcomes (task performance and job satisfaction) of organizational socialization that may result from newcomers’ self-efficacy elicited by receiving mentoring (Kammeyer-Mueller and Wanberg, 2003).

To be specific, we contend that occupational self-efficacy is crucial for leading to newcomers’ favorable task-related outcomes (e.g., task mastery, task performance, job satisfaction), where task mastery reflects the extent to which one’s capability to successfully fulfill job demands (Kammeyer-Mueller and Wanberg, 2003) and task performance refers to supervisors’ appraisal of newcomers’ performance at work. That is, we argue that newcomers with high levels of self-efficacy in the occupational context are prone to show great confidence and persistence in their abilities to perform tasks (Spurk and Abele, 2014), undertake challenging tasks (Sexton et al., 1992), and proactively seek ways to resolve problems they encounter at work, which subsequently enhancing their task mastery and facilitate them to accomplish their performance satisfactorily (Guarnaccia et al., 2018). Furthermore, job satisfaction captures the extent to which an employee’s expectations and psychological needs are being met (Gruneberg, 1979; Aziri, 2011). In this sense, newcomers who believe that they can master work-related challenges may boost their experience of high psychological wellbeing and sense of accomplishment at work (Avey et al., 2010), which likely triggers a feeling of satisfaction with their job. In addition, existing research also has provided empirical supports for the significant impacts of employees’ occupational self-efficacy on key career outcomes such as role clarity, work engagement, task performance, and job satisfaction (Hirschi, 2012; Spurk and Abele, 2014; Jiang et al., 2016; Alon et al., 2021).

As such, we further postulate newcomers’ self-efficacy in the social interaction context as a critical antecedent to aspects of their social outcomes (e.g., social integration and job satisfaction). In particular, social integration refers to the extent to which one’s feeling of attachment and inclusion in the current organization (Morrison, 2002), indicating the fitness of newcomers in this group. Newcomers with high levels of social self-efficacy are confident in situations involving social contact (Luo et al., 2018) and tend to take initiatives to develop relationships with other group members and improve their social environment (Caspi and Bern, 1990; Gu et al., 2014). Such positive interactions with others help newcomers engage in socially acceptable behaviors and build favorable reputations among coworkers, which facilitate their social integration and enhance their feelings of satisfaction with their job (Montani et al., 2019). Similarly, several other researchers have highlighted the facilitative role social self-efficacy played in predicting socialization outcomes in the workplace social domains such as job-related affective wellbeing, job satisfaction, and social adjustment (Fan et al., 2013; Romera et al., 2016; Luo et al., 2018). To summarize, we propose the following hypotheses:

*Hypothesis 3: Newcomer occupational self-efficacy is positively related to (a) task mastery, (b) task performance, and (c) job satisfaction.*

*Hypothesis 4: Newcomer social self-efficacy is positively related to (a) job satisfaction and (b) social integration.*

We further suggest that receiving career mentoring, by improving newcomers’ social cognitions of occupations may promote their occupational self-efficacy beliefs, which, in turn, could enhance their task mastery, task performance, and job satisfaction. Simultaneously, we also argue that newcomers’ social self-efficacy may act as a mediating mechanism, transmitting the positive effects of psychosocial mentoring on newcomers’ job satisfaction and social integration. Taken together, we advance the following hypotheses:

*Hypothesis 5: Newcomer occupational self-efficacy mediates the relationship between career mentoring and (a) task mastery, (b) task performance, and (c) job satisfaction.*

*Hypothesis 6: Newcomer social self-efficacy mediates the relationship between psychosocial mentoring and (a) job satisfaction and (b) social integration.*

### The moderating role of newcomers’ learning adaptability

The social cognitive career theory emphasized that employees’ learning experience in career development is influenced by not only environmental factors but also employee characteristics (Lent et al., 1994; Lopez et al., 1997). That is, employee characteristics can determine actual contextual supports they receive from the organization to some extent and further alter employees’ interpretation and acceptance of these supportive treatments (Cullen et al., 2014). Combined with the newcomer context in our study, this proposition is also aligned with Wang et al. (2021) suggesting that newcomers’ differences in learning adaptability may influence their attitudes about integrating external information and supports during the socialization process. Learning adaptability, reflecting the willingness to learn to adapt and stay current in the profession (Wang et al., 2021), determines the success of newcomer learning-oriented adaptation during the work role transition process to some extent (Wang et al., 2021). Accordingly, we suggest that the intensity of the relationship between specific mentoring and newcomers’ domain-specific self-efficacy may be contingent upon the levels of learning adaptability.

Drawing on social cognitive career theory, learning adaptability can be regarded as an important personal characteristic related to the formation of employee personal cognition factors since it can directly affect the employee’s learning experience (Wang et al., 2011; Međugorac et al., 2020). As such, in the current study, newcomers who have stronger learning adaptability are more likely to absorb the functional technology and skills provided by their mentors and improve the effectiveness of their certain learning experience, ultimately developing positive self-cognition accordingly. To be specific, in the case of receiving career mentoring, this would include learning the skills essential to complete task-related aspects of work (Craig et al., 2013). Newcomers with strong learning adaptability tend to exemplify some traits such as being proactive, resourceful, and resilient (Cullen et al., 2014). Thus, when they have received mentors’ guidance on career, the learning-related initiative may facilitate their integration and assimilation of expertise during the learning process and thus improve their confidence in dealing with task-related issues, that is, occupational self-efficacy. Likewise, when newcomers have received psychosocial mentoring, high learning-oriented adaptability may allow them to comprehend the social and psychological cues provided by mentors and gain an accurate grasp of interpersonal skills (Cullen et al., 2014), thereby may be well positioned to promoting their self-efficacy in social contact.

Moreover, previous research on learning adaptability also provides supports for the inferences mentioned before. For example, Liu et al. (2014) and Boulamatsi et al. (2020) recognized that at the early stages of career, employees with strong learning adaptability are prone to adopt active strategies to adjust themselves in the learning process, which can facilitate them to master new skills and achieve the balance in the new environment. As such, in the context of mentoring, newcomers high in learning adaptability likely take initiatives to keep themselves in harmony with the new environment by gaining and assimilating new specialized expertise from mentors’ specific guidance, which also contributes to boosting their domain-specific self-efficacy. In sum, we propose the following hypotheses:

*Hypothesis 7: Newcomer learning adaptability strengthens the positive effect of career mentoring on occupational self-efficacy.*

*Hypothesis 8: Newcomer learning adaptability strengthens the positive effect of psychosocial mentoring on social self-efficacy.*

## Methods

### Sample and procedure

Our sample consisted of newcomers (employees who are within the first 6 months of joining their current company) and their supervisors from two high-tech companies located in the northern part of China, one specializing in household appliance manufacturing and the other in battery manufacturing. These participants were employed in different departments, including R&D, engineering, production, operations, marketing, and manufacturing. During the preliminary interviews with several newcomers and supervisors, we found that mentoring was prevalent in both companies, especially for newcomers, which was comprehensive and varied. Most newcomers indicated that such mentoring facilitated them to integrate into the current organization. Thus, we believe that these companies are appropriate contexts to test the associations between different forms of mentoring and newcomer socialization.

Before the formal survey began, under the assistance of the human resources department, we sent an email to newcomers introducing the purpose and procedures of our investigation and inquiring about their intention to participate. Finally, we received 287 positive responses and further included them and their supervisors as participants, matching each of them with a four-digit code. To minimize the potential common method bias and relieve participantse human resources department, we sent an email to newcom. We maintained a 1-month interval between each wave of data collection. At time 1, newcomers were asked to report career and psychosocial mentoring, learning adaptability, and demographics. At time 2, newcomers were instructed to report their occupational and social self-efficacy, social exchange relationship with supervisor, and perceived supervisor support. At time 3, we asked newcomers to self-report their task mastery and job satisfaction, while their supervisors were required to rate newcomers’ task performance and social integration, with each supervisor rating an average of 1.78 newcomers.

Finally, 157 newcomers and 88 supervisors of the total participants returned surveys with completed and matched data, representing an overall response rate of 55.70%. Of the valid sampled newcomers, 61.15% were men, and 83.44% had attained a bachelornse rate of 55.70%. Of the job satisfaction, years old (*SD* = 2.63). The average organizational tenure and previous working experience of newcomers were 2.77 months (*SD* = 1.45) and 9.66 months (*SD* = 11.31), respectively.

### Measures

All substantive variables were measured on a seven-point Likert scale (1 = ‘strongly disagree’, 7 = ‘strongly agree’), unless otherwise specified. Following the translation-back-translation procedure suggested by Brislin (1980), the original English items were translated into Chinese.

***Career and psychosocial mentoring.*** Career and psychosocial mentoring were measured with a 14-item scale developed by Viator (2001) and Dreher and Ash (1990). Given that newcomers may receive developmental supports from multiple mentors, we asked them to consider their actual experience within the current company and to rate the extent to which “higher-ranking individuals who had advanced experience and knowledge” have provided career and psychosocial mentoring to them (Bozionelos et al., 2011). In the scale, six items measure career mentoring (e.g., “… recommended or supported you in obtaining assignments which offered opportunities to learn new skills, or develop expertise in a specific area”; α = 0.904), and eight items reflect psychosocial mentoring (e.g., “… conveyed empathy for the concerns and feelings you have discussed”; α = 0.923).

***Occupational and social self-efficacy.*** Occupational and social self-efficacy were measured using items from the social and emotional self-efficacy scale developed by Loeb et al. (2016). Occupational self-efficacy was assessed using six items (α = 0.819), such as “When I am confronted with a problem in my job, I can usually find several solutions.” Social self-efficacy was prompted by “To what extent you have confidence in your ability to …” and including 5 items (α = 0.914) such as “Start a conversation at work with someone you don’t know very well.” Response choices ranged from 1 (no confidence at all) to 7 (complete confidence).

***Task mastery.*** Task mastery was measured with the five-item performance proficiency scale developed by Chao et al. (1994). A sample item is “I understand what all the duties of my job entail” (α = 0.831).

***Task performance.*** Task performance was captured with a three-item scale developed by Li et al. (2011). A sample item is “This newcomer performs the tasks that are expected as part of the job” (α = 0.772).

***Job satisfaction.*** Job satisfaction was measured with a three-item scale developed by Cammann et al. (1983). A sample item is “All in all, I am satisfied with my job” (α = 0.716).

***Social integration.*** Social integration was measured with a three-item scale developed by Kim et al. (2009). Supervisors were asked to rate the extent to which newcomers performed well in their interpersonal relationships in the workplace (α = 0.722), such as “socializing with coworkers.” Response choices ranged from 1 (needs much improvement) to 7 (excellent).

***Learning adaptability.*** Learning adaptability was measured using five items (α = 0.942) from the Ployhart and Bliese (2006) scale, including “I quickly learn new methods to solve problems.”

***Control variables.*** Consistent with prior socialization and mentoring research, this study included newcomers’ gender, age, education, organizational tenure, previous working experience, and company as control variables. In addition, Ou et al. (2018) showed that the high-quality exchange relationship between newcomer and supervisor might facilitate the newcomer to shape perceived insider status, which might be conducive to socialization outcomes. Thus, we included newcomer–supervisor exchange as the control variable using a seven-item scale developed by Graen and Uhl-Bien (1995). A sample item is “How well does your supervisor recognize your potential? (1 = not at all to 5 = fully, α = 0.908).” Perceived supervisor support was also assigned to control for potential confusion, where differences in supervisor support might affect newcomer adjustment (Dufour et al., 2021). It was measured with an eight-item scale developed by Eisenberger et al. (2002). A sample item is “My supervisor is willing to help me when I need a special favor” (α = 0.932).

## Results

### Preliminary analyses

Table 1 presents the means, standard deviations, and correlations of all variables. Given the nested nature of our data, we then calculated the intra-class correlation (ICC) coefficients for two variables that were rated by supervisors. The ICC(1) values of newcomer task performance and social integration were 0.002 and 0.043, respectively, which were lower than its cutoff value of 0.12 (Ployhart et al., 2006). The ICC(2) values of newcomer task performance and social integration were 0.004 and 0.074, respectively. Overall, these results indicated that the nested data structure did not affect the relationships in our study.

**TABLE 1:** Means, standard deviations, and correlations of variables.

Variables	Mean	*SD*	1	2	3	4	5	6	7	8	9	10	11	12	13	14	15	16
1. Age	27.885	2.634																
2. Gender	0.611	0.489	–0.065															
3. Education	1.975	0.554	0.551**	0.153														
4. Organizational tenure	2.771	1.445	–0.012	0.055	–0.095													
5. Previous working experience	9.656	11.308	0.501**	–0.019	–0.177*	0.023												
6. Company	1.395	0.490	–0.009	–0.185*	0.108	–0.143	–0.129											
7. CM	4.535	1.309	0.005	–0.022	0.069	0.094	0.050	0.090										
8. PSM	4.638	1.332	–0.017	0.042	0.056	0.185*	–0.021	0.186*	0.185*									
9. OS	4.383	1.198	0.097	–0.076	0.164*	0.050	–0.123	0.215**	0.317**	0.040								
10. SS	4.439	1.648	–0.110	–0.095	–0.026	0.152	–0.090	0.106	–0.048	0.374**	0.146							
11. LA	5.487	1.582	–0.124	0.055	0.010	0.107	–0.176*	0.053	0.004	0.090	0.200*	0.000						
12. TM	5.050	1.229	0.007	0.002	0.053	0.052	–0.137	0.133	0.028	0.136	0.458**	0.142	0.266**					
13. TP	4.713	1.215	–0.189*	–0.084	–0.046	–0.026	–0.175*	0.177*	0.144	–0.026	0.290**	–0.082	0.399**	0.263**				
14. JS	4.962	1.011	0.036	–0.155	0.117	0.019	–0.132	0.052	0.046	0.027	0.392**	0.321**	0.150	0.225**	0.289**			
15. SI	4.694	1.079	–0.091	–0.041	–0.035	0.099	–0.093	–0.065	–0.003	0.044	0.143	0.274**	0.167*	0.065	0.064	0.242**		
16. NSX	3.611	1.049	–0.273**	–0.077	–0.160*	0.139	–0.205*	0.060	0.057	0.072	0.163*	0.211**	0.145	0.261**	0.200*	0.233**	0.183*	
17. PSS	4.793	1.407	–0.142	–0.006	–0.135	0.158*	–0.071	–0.015	0.100	0.085	0.168*	0.227**	0.059	0.232**	0.278**	0.232**	0.085	0.133

*N* = 157. Gender: 0 = female, 1 = male; education: 1 = bachelorucation: 1 experience = masterorucation: 1 = doctororucation: organizational tenure and previous working experience were measured by months. CM, career mentoring; PSM, psychosocial mentoring; OS, occupational self-efficacy; SS, social self-efficacy; LA, learning adaptability; TM, task mastery; TP, task performance; JS, job satisfaction; SI, social integration; NSX, newcomerntegration; NSXTMy; SSxpe, perceived supervisor support. *p < 0.05, **p < 0.01 (two-tailed tests).

### Confirmatory factor analysis

Following the suggestion proposed by Little et al. (2013) and Kline (2016), we used item parceling to form three parcels each for all latent variables (apart from task performance, job satisfaction, and social integration that we measured with the three-item scales), which was conducive to keep adequate indicator-to-sample size ratio. Each parcel was formed by randomly combining assigned items. Then, we conducted a confirmatory factor analysis to test the fitness of the integrated model. As demonstrated in Table 2, the hypothesized nine-factor model was a better fit to the data, with χ^2^/*df* = 1.152, *p* < 0.001; CFI = 0.981, TLI = 0.977, RMSEA = 0.031, and SRMR = 0.050, than all the alternative models. Thus, the results indicated discriminant validity for our focal variables.

**TABLE 2:** Results of confirmatory factor analysis.

Model	χ^2^	*df*	χ^2^/*df*	CFI	TLI	RMSEA	SRMR
Hypothesized nine-factor model	331.843***	288	1.152	0.981	0.977	0.031	0.050
**Eight-factor models**							
Combining JS and SI	412.014***	296	1.392	0.950	0.941	0.050	0.061
Combining TM and TP	473.675***	296	1.600	0.924	0.910	0.062	0.078
Combining CM and PSM	698.077***	296	2.358	0.828	0.796	0.093	0.097
Combining OS and SS	704.484***	296	2.380	0.825	0.793	0.094	0.100
Seven-factor model (combining TM, TP, and JS)	579.497***	303	1.913	0.882	0.863	0.076	0.091
**Six-factor models**							
Combining TM, TP, JS, and SI	684.396***	309	2.215	0.839	0.818	0.088	0.102
Combining CM, PSM, OS, and SS	1,305.334***	309	4.224	0.574	0.516	0.143	0.154
Four-factor model (CM and PSM vs. OS and SS vs. LA vs. TM, TP, JS, and SI)	1,373.093***	318	4.318	0.549	0.502	0.145	0.145
Two-factor model (combining newcomer-rated variables vs. supervisor-rated variables)	2,049.325***	323	6.345	0.261	0.197	0.185	0.165
One-factor model (combining all variables)	2,126.685***	324	6.564	0.229	0.164	0.188	0.164

*N* = 157. CFI, the comparative fit index; TLI, Tucker-Lewis index; RMSEA, root mean square error of approximation; SRMR, standardized root mean square residual. ***p < 0.001.

### Hypotheses testing

**FIGURE 1:**
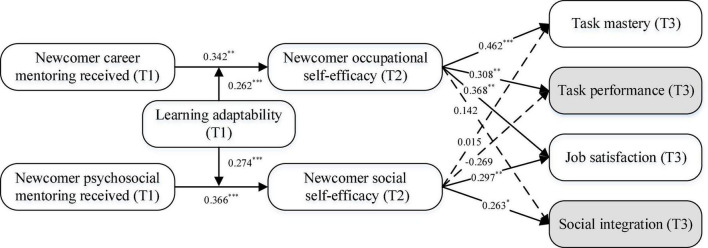
Structural equation model. *N* = 157. Standardized parameter estimates of the hypothesized model using structural equation modeling (*p < 0.050, ^**^p < 0.010, ^***^p < 0.001). “T1”, “T2”, and “T3” refer to the three data collection waves. Data were collected from supervisors are in shaded rounded rectangles, and other data from newcomers are in a rounded rectangle without shade.

We performed the structural equation modeling using the maximum-likelihood estimation method in Mplus 8.3 to calculate standardized path coefficients. As shown in Figure 1, newcomer career mentoring was significantly related to occupational self-efficacy (*B* = 0.342, *SE* = 0.104, 95% CI = [0.138, 0.546]), whereas newcomer psychosocial mentoring significantly predicted social self-efficacy (*B* = 0.366, *SE* = 0.088, 95% CI = [0.193, 0.539]), as such, hypotheses 1 and 2 were both supported. Newcomer occupational self-efficacy was significantly and positively related to task mastery (*B* = 0.462, *SE* = 0.092, 95% CI = [0.281, 0.642]), task performance (*B* = 0.308, *SE* = 0.095, 95% CI = [0.123, 0.494]), and job satisfaction (*B* = 0.368, *SE* = 0.121, 95% CI = [0.132, 0.604]), thereby supporting hypotheses 3a, 3b, and 3c. Likewise, significant positive associations existed between newcomer social self-efficacy and job satisfaction (*B* = 0.297, *SE* = 0.107, 95% CI = [0.086, 0.507]), as well as social integration (*B* = 0.263, *SE* = 0.115, 95% CI = [0.037, 0.488]), which provide supports for hypotheses 4a and 4b. Furthermore, the results of the bootstrapping test revealed that the indirect effects of newcomer career mentoring on task-related outcomes through occupational self-efficacy were significantly positive (*B* = 0.158, *SE* = 0.050, 95% CI = [0.059, 0.256], for task mastery; *B* = 0.105, *SE* = 0.045, 95% CI = [0.017, 0.194], for task performance; *B* = 0.126, *SE* = 0.057, 95% CI = [0.015, 0.237], for job satisfaction), supporting hypotheses 5a, 5b, and 5c. Similarly, the positive indirect effects of newcomer psychosocial mentoring on social outcomes *via* social self-efficacy were significant (*B* = 0.109, *SE* = 0.049, 95% CI = [0.013, 0.204], for job satisfaction; *B* = 0.096, *SE* = 0.048, 95% CI = [0.003, 0.190], for social integration), supporting hypotheses 6a and 6b.

**FIGURE 2:**
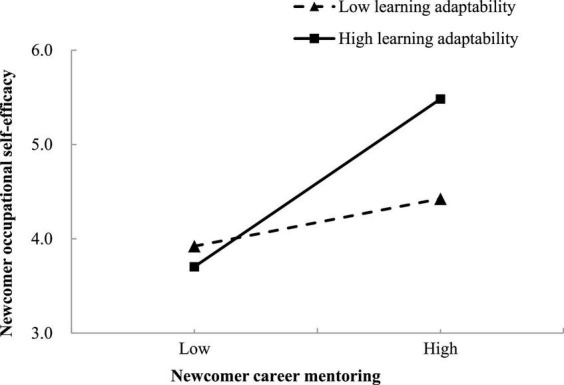
Interaction effect between newcomer career mentoring and learning adaptability on occupational self-efficacy.

**FIGURE 3:**
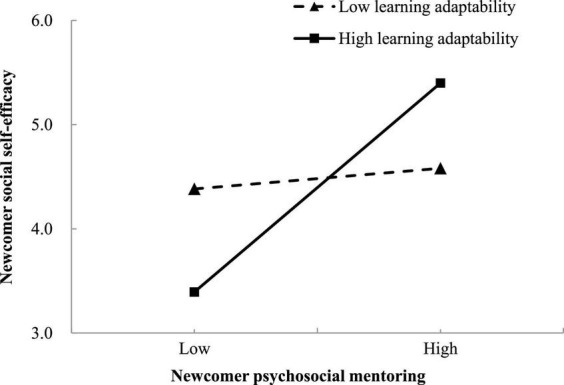
Interaction effect between newcomer psychosocial mentoring and learning adaptability on social self-efficacy.

Then, the results also demonstrated the significant interaction effect of newcomer career mentoring and learning adaptability on occupational self-efficacy (*B* = 0.266, *SE* = 0.052, 95% CI = [0.164, 0.367]), as well as the significant interaction effect of newcomer psychosocial mentoring and learning adaptability on social self-efficacy (*B* = 0.274, *SE* = 0.072, 95% CI = [0.134, 0.415]). Moreover, the results of a simple slope test plotted in Figure 2 presented that for newcomers who possess higher learning adaptability (+1 SD above the mean), career mentoring was more strongly and positively associated with occupational self-efficacy (*B* = 0.622, *SE* = 0.105, 95% CI = [0.417, 0.828]); for those with lower learning adaptability (–1 SD below the mean), such association was weaker but still significant (*B* = 0.176, *SE* = 0.073, 95% CI = [0.033, 0.320]). Likewise, Figure 3 displayed that the positive effect of psychosocial mentoring and social self-efficacy was significantly stronger (*B* = 0.652, *SE* = 0.121, 95% CI = [0.414, 0.890]) when newcomers have higher learning adaptability, whereas the effect was weaker and not significant (*B* = 0.064, *SE* = 0.122, 95% CI = [–0.174, 0.303]) when newcomers’ learning adaptability was low. Accordingly, hypotheses 7 and 8 were supported.

## Discussion

Using the social cognitive career theory as a theoretical foundation, we developed and examined an integrated model to reveal the mechanisms through which different forms of mentoring affect newcomer socialization outcomes. In particular, our findings indicated that newcomer occupational self-efficacy mediated the positive relationships between career mentoring and task mastery, task performance, and job satisfaction. Newcomer social self-efficacy mediated the positive relationships between psychosocial mentoring and job satisfaction as well as social integration. Furthermore, our findings showed that newcomer learning adaptability strengthened the relationship between career mentoring and occupational self-efficacy, as well as the linkage between psychosocial mentoring and social self-efficacy. These findings paved the way for the understanding of how and when different types of mentoring promote certain indicators of socialization.

### Theoretical implications

Our study provides several theoretical contributions to the research on mentoring and newcomer socialization. First, our study enriches mentoring literature by linking different forms of mentoring and newcomer domain-specific socialization outcomes. Although extant studies have shown that mentoring can facilitate mentee socialization (Thomas and Lankau, 2009; Yang et al., 2013; Son, 2016; Gazaway et al., 2019; Cai et al., 2021), limited studies have finely investigated when and why receiving different forms of mentoring may facilitate newcomer task-related and social indicators of socialization during organizational entry (Allen et al., 2017). Our research indicates that career and psychosocial mentoring can elicit newcomer occupational and social self-efficacy, which are further positively related to their occupational and social outcomes, respectively. In so doing, we also answer the calls to investigate how specific mentoring differentially relates to mentee socialization outcomes (Baranik et al., 2010; Yang et al., 2013).

Second, our study sheds light on domain-specific self-efficacy research in terms of how career and psychosocial mentoring have differential effects on newcomers’ occupational and social self-efficacy. Researchers have previously suggested that mentoring receiving can promote mentee’s general self-efficacy (Eby et al., 2013; St-Jean et al., 2018; Kao et al., 2019). However, relatively little attention has been focused on exploring how different forms of mentoring differentially affect mentees’ domain-specific self-efficacy. Building on the social cognitive career theory, our findings provide empirical evidence that receiving career and psychosocial mentoring can enhance newcomers’ occupational and social self-efficacy, respectively, ultimately contributing to positively affecting their occupational and social outcomes. Our study also responds to the call proposed by Loeb et al. (2016) to explore the antecedents and the consequences of different domain-specific self-efficacy.

Third, by introducing learning adaptability as a learning-related personal characteristic into mentoring literature, our study highlights learning adaptability as a boundary condition for the influence of mentoring on newcomers’ self-efficacy from the cognitive perspective. More specifically, our findings demonstrate that the positive influences of career and psychosocial mentoring on newcomers’ occupational and social self-efficacy are more significant when newcomers possess stronger learning adaptability. These findings not only answer the calls for more attention to boundary conditions such as mentees’ characteristics of the associations between mentoring and mentees’ work outcomes (Pan et al., 2011) but also follow Son’s (2016) suggestion on testing regarding the effects of mentees’ learning-related factors on their further work outcomes. Our emphasis on the role of mentees’ learning adaptability enriches and expands the research on mentoring.

### Practical implications

Our study also offer insights into management practices. First, our study reveals that receiving different forms of mentoring is conducive to facilitating newcomers’ socialization in corresponding domains. Thus, we suggest that newcomers are encouraged to seek senior colleagues who possess advanced experience and knowledge and proactively build mentoring relationships with them, which can provide many benefits to socializing. Simultaneously, given limits on the mentors’ resources, there may be barriers for newcomers to establish mentoring relationships (Yang et al., 2013). Thus, experienced employees should be trained on how they could tactically provide effective mentoring (e.g., acceptance, counseling, and friendship) to meet newcomers’ personal needs in different socialization contexts. In addition, rewarding collaborative behaviors through performance management systems may be useful in motivating experienced employees to offer informal mentoring.

Second, our study underlines the importance of domain-specific self-efficacy in linking different forms of mentoring and newcomer occupational and social outcomes. Fan et al. (2013) stated that employees’ self-efficacy in the workplace is changeable and subject to external influences. Thus, we suggest that organizations should target certain domains, where newcomers show strengths or shortcomings and further make corresponding arrangements to boost their specific self-efficacy. For instance, organizations can use diagnostic tools to evaluate employees’ self-efficacy during the early socialization phase (Loeb et al., 2016). Then, according to the evaluation results, organizations can specially conduct occupational or social skills training and coaching for those who need to improve occupational self-efficacy or social self-efficacy, respectively, which, in turn, accelerates the process of newcomer adjustment.

Third, our findings of the moderating role of learning adaptability suggest that newcomers with strong learning adaptability can amplify the positive impacts of mentoring on their self-efficacy, ultimately contributing to accomplishing their socialization process. As such, this study emphasizes the value of learning adaptability and its facets for the selection, mentoring, and the development of newcomers in the organizations. Accordingly, HR managers are encouraged to select employees with high levels of learning adaptability in recruitment. In addition, organizations should strive to cultivate newcomers’ problem-solving mentality and foster their positive framing of the work situation, maximizing their learning adaptability potential (Boulamatsi et al., 2020).

### Limitations and future research

Despite our efforts, our study has several potential limitations. First, consistent with several previous research on mentoring (Menges, 2016; Lewis et al., 2017), we tested all hypotheses by using multisource and paired data to alleviate the influence of the common method variance. The results derived through confirmatory factor analysis further suggested that all variables were distinct from each other. Nonetheless, the effects of the common method variance on the findings cannot completely be ruled out, especially on interpreting causal influences. Therefore, using a longitudinal design in future research is necessary to reduce such concerns directly and test causality in the mediation process (Preacher, 2015).

In addition, since we could not rule out other potential mediating mechanisms that can also link mentoring to newcomer socialization, much work remains to be done in this respect. For example, employing self-determination theory, Janssen et al. (2020) suggested that mentoring relationships have far-reaching implications for mentee careers by meeting their basic needs for autonomy, competence, and relatedness. Thus, it is worth further considering that receiving career and psychosocial mentoring may meet mentees’ psychological needs in the corresponding domain and then facilitate them to socialize. As such, future research ought to explore different mediation processes to comprehensively explain the process by which different forms of mentoring differentially affect mentee socialization.

Finally, our study only explores mentee characteristics (i.e., learning adaptability) as the boundary condition for the linkage between mentoring and socialization outcomes. Future empirical studies could consider alternative contextual explanations to further expand the mentoring literature. For instance, mentoring scholars have demonstrated that not all mentorships are constructive. The effectiveness of mentorship varies according to relationship quality and mentees’ trust in mentors (Lyons and Perrewe, 2014; Son and Kuchinke, 2016; Kwan et al., 2021). Mentees who perceive low-quality mentorship or lack trust in their mentors are inclined to report being less satisfied with mentoring they received (Xu and Payne, 2014), which may impair the positive impacts of mentoring. Thus, future researchers can explore mentorship characteristics (i.e., relationship quality and trust in mentors) as possible boundary conditions in efforts to account for when receiving different forms of mentoring could promote mentees’ domain-specific self-efficacy.

## Conclusion

Drawing on the social cognitive career theory, our study uncovered the differential influences of different types of mentoring on boosting newcomers’ organizational socialization by introducing their self-efficacy in the corresponding domain as the potential mechanisms. Moreover, the findings in our study contribute to the existing literature by revealing that the extent to which different types of mentoring stimulate newcomers’ specific self-efficacy was moderated by their learning adaptability. Altogether, these findings provided a comprehensive picture for scholars and managers to understand the specialized roles of mentoring on newcomer adjustment.

## Data availability statement

The raw data supporting the conclusions of this article will be made available by the authors, without undue reservation.

## Ethics statement

The studies involving human participants were reviewed and approved by the Ethical Review Board of Beijing Jiaotong University. The patients/participants provided their written informed consent to participate in this study.

## Author contributions

HD: conceptualization, methodology, investigation, data curation, and writing—original draft preparation. WenbW: investigation and writing—reviewing and editing. YZ, ZY, and HX: writing—reviewing and editing. WenW: data curation. All authors have read and agreed to the submitted version of the manuscript.
